# Spread of Cantagalo Virus to Northern Brazil

**DOI:** 10.3201/eid1507.081702

**Published:** 2009-07

**Authors:** Maria Luiza G. Medaglia, Leila Cristina G.D. Pessoa, Elisabeth R.C. Sales, Tânia R.P. Freitas, Clarissa R. Damaso

**Affiliations:** Instituto de Biofísica Carlos Chagas Filho, Rio de Janeiro, Brazil (M.L.G. Medaglia, C.R. Damaso); Agência de Defesa Agropecuária do Estado do Tocantins, Tocantins, Brazil (L.C.G.D. Pessoa, E.R.C. Sales); Laboratório Nacional Agropecuário, Pedro Leopoldo, Minas Gerais, Brazil (T.R.P. Freitas)

**Keywords:** Cantagalo virus, vaccinia virus, poxvirus, zoonosis, zoonoses, viruses, virus spread, letter

**To the Editor:** *Cantagalo virus* (CTGV) is a strain of *vaccinia virus* (VACV; *Poxviridae*) that was isolated from pustular lesions on dairy cattle and dairy workers in Rio de Janeiro State, Brazil, in 1999 (*1*). Subsequently, similar lesions caused by poxviruses have been reported in cattle and humans in all 4 states of the southeast region of Brazil and in Goiás State in central-western Brazil (Technical Appendix, panel A, (*2*–*7*). Etiologic agents were VACV strains, most of which were genetically related to CTGV, such as Araçatuba and Passatempo viruses (*2*,*4*), with the exception of Guarani P1 virus, which was isolated in Minas Gerais State in 2001 and is phylogenetically close to VACV strain WR (*5*). Reisolation of Guarani P1 virus has not been reported. All VACV isolates related to CTGV share 2 molecular signatures: an 18-nt deletion in the A56R gene, which encodes viral hemagglutinin (*1*–*7*), and a 15-nt deletion in K2L gene, which encodes serine protease inhibitor-3 (*8*,*9*). Other VACV strains unrelated to CTGV were isolated from rodents in Brazil before 1999, but reisolation of these viruses has not been described (8). Although CTGV-like disease has not been reported in the northern, northeastern, and southern regions of the country, rapid interstate spread of CTGV infection is of concern. We report an episode of CTGV infection in Tocantins State, northern Brazil (Technical Appendix, panel A).

In September 2008, teat and udder lesions were found on 15 of 356 febrile (39.5°C–40°C) cattle on a dairy farm in the municipality of Muricilândia. Small papules progressed to vesicles and pustules (Technical Appendix, panel B), which usually healed in 3–4 weeks. New lesions subsequently appeared on previously healthy cows on the same farm, and muzzle lesions developed on suckling calves. Dairy workers reported fever and lesions on their hands and neck. The farm was quarantined for 3 weeks until the condition was diagnosed.

Four scab samples were sent for virus identification by PCR. Parts of the samples were used to infect BSC-40 cells and for DNA isolation by phenol-chloroform extraction, as described (*6*). After 48 hours, a strong cytopathic effect suggested poxvirus infection. The PCR used unambiguously differentiates CTGV-related infections from other orthopoxvirus diseases, including cowpox virus and several VACV strains (*6*). The reverse primer targets nucleotide sequences flanking the deletion signature of the hemagglutinin gene from CTGV-related viruses. Therefore, a specific annealing site for the reverse primer is produced when these external sequences are contiguous, as occurs in CTGV (*6*).

The full-length hemagglutinin gene (≈900 bp) was detected in all clinical isolates and in the control DNA samples from CTGV, VACV strain WR, and cowpox virus strain Brighton-red (Technical Appendix, panel C). Nevertheless, when we used the primers specific for CTGV detection, only CTGV and the 4 isolates were positive, generating 714-bp fragments, which indicated CTGV as the etiologic agent. In late November, the disease was reported in 9 cattle in Santa Fé do Araguaia, a municipality 12 km west of Muricilândia. Those samples were also positive for CTGV by PCR (data not shown).

For phylogenetic inference, we used DNA from the isolate MU-07 to sequence the genes A56R (927 bp), C7L (453 bp) that encodes a host-range virulence factor, and K2L (1095 bp); primers aligned externally to the open reading frames. PCR and sequencing were performed as described elsewhere (*1*). Sequences were deposited in GenBank (accession nos. FJ545689, FJ545688, and FJ545687, respectively). Nucleotide identities in relation to CTGV sequences were 99.8% (A56R), 100% (C7L), and 100% (K2L). Both A56R and K2L genes had deletions considered to be molecular signatures for Brazilian VACV related to CTGV. Phylogenetic inference of the concatenated nucleotide dataset of 27 orthopoxviruses shows that the causative agent grouped with other Brazilian VACV related to CTGV (Technical Appendix, panel D).

We consider the etiologic agent of the infection in Tocantins State to be a CTGV isolate, indicating spread of CTGV infection to northern Brazil. This spread could reflect interstate propagation of the virus due to movement of animals or people, which is particularly intense at the southern border with Goiás State (location of the nearest CTGV outbreak) (*3*). Nevertheless, no epidemiologic data are available to support a relationship between these episodes. The Agency for Animal Health Defense of Tocantins State has not been previously notified of clinical suspicion of poxvirus infection in dairy herds.

Another concern is spread of the virus to water buffalo, which account for a growing farming industry in Brazil, specifically in the northern states (www.ibge.gov.br/english). Infected buffalo have not yet been reported in Brazil, but the establishment of VACV strains in buffaloes has long been reported in India; economic losses have been substantial (9). Therefore, a careful survey should be conducted to evaluate dissemination of the virus to other states and species in the Amazon region.

## Supplementary Material

Technical Appendix Spread of Cantagalo Virus to Northern Brazil
