# Supplementary material for: Spread of Cantagalo Virus to Northern Brazil
Source: Emerg Infect Dis. 2009 Jul;15(7):1142–3. doi: 10.3201/eid1507.081702 (PMC2744230; doi:10.3201/eid1507.081702)
Supplement: Technical Appendix — Spread of Cantagalo Virus to Northern Brazil [file 08-1702-Techapp.pdf]

Spread of Cantagalo Virus to Northern Brazil

Technical Appendix

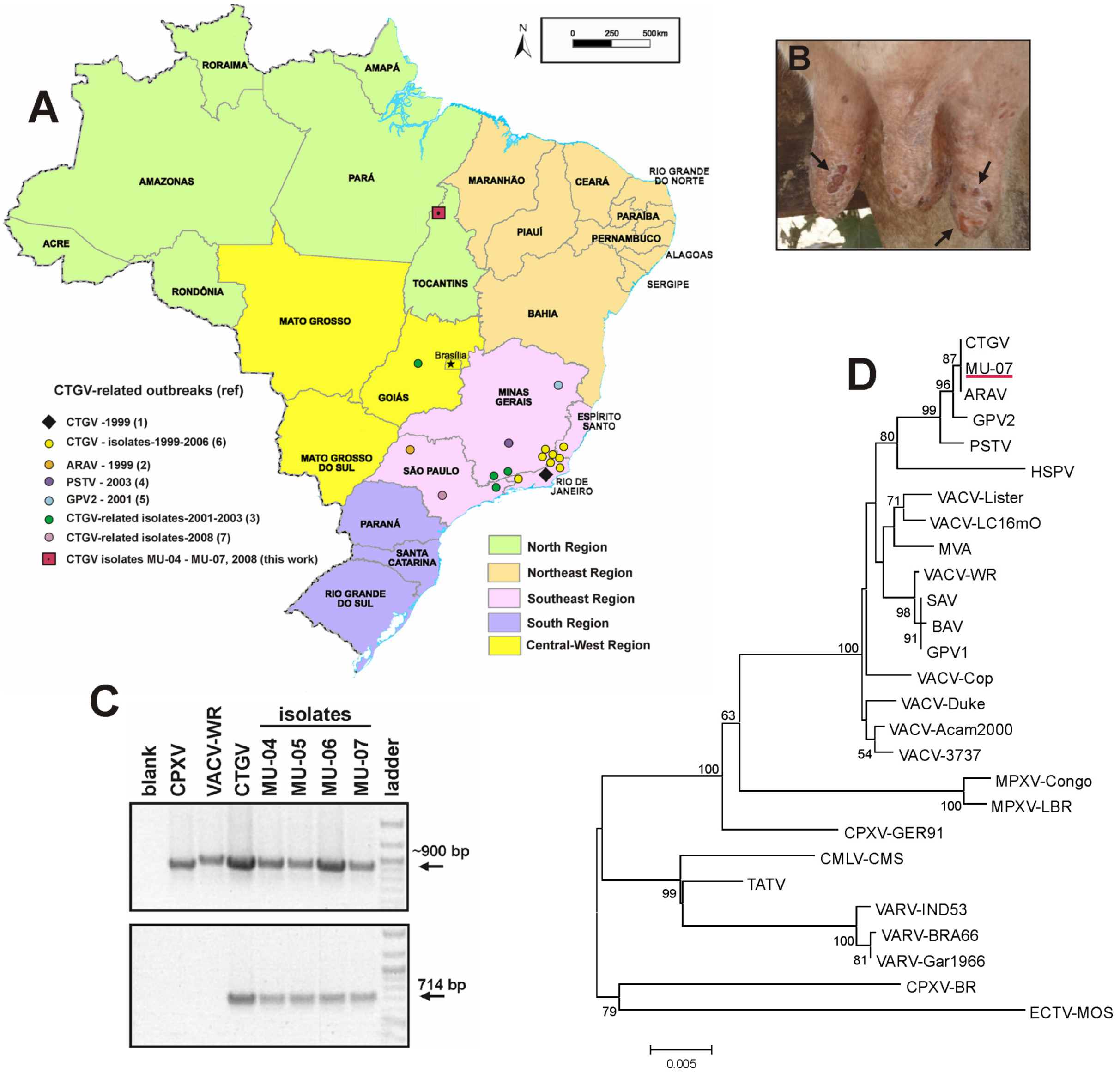

Figure. A) Geographic regions and states (from Instituto Brasileiro de Geografia e Estatística, with permission). Locations of Cantagalo-like episodes reported since 1999 are specified. B) Virus lesions (scabs) on cow teats after infection with isolate MU-07. C) PCR analysis of full-length A56R gene (upper panel) and fragment of A56R gene with the 18-nt deletion (lower panel). D) Concatenated alignment of isolate MU-07 and 26 orthopoxviruses based on the individual alignments for A56R, C7L, and K2L,

aligned by using ClustalX version1.81 (www.clustal.org). Phylogeny inference was performed by using MEGA 4 (www.megasoftware.net) opting for the neighbor-joining method and Kimura 2-parameter model of substitution. Numbers indicate the percentage of bootstrap support from 1,500 replicates. Values >50% are shown. Scale bar represents nucleotide changes. Virus species and GenBank accession numbers are as follows: VACV strains: ARAV (Araçatuba virus; AY523994, EF051277, EF175987), CTGV (Cantagalo virus; AF229247, EF488959, EU528619), GPV2 (Guarani P2 virus; DQ206437, EF051280, EF175988), PSTV (Passatempo virus; DQ070848, EF051282, EF175989), HSPV (horsepox virus; DQ792504), Lister (AY678276), LC16mO (AY678277), MVA (DQ983236), WR (NC_006998), GPV1 (Guarani P1 virus; DQ206436, EF051279, EF175991), BAV (BeAn 58058; DQ206442, EF051278, EF175990), SAV (SPAn 232; DQ222922, EF051283, EF175992), Cop (Copenhagen; M35027), Duke (DQ439815), Acam2000 (AY313847), 3737 (DQ377945); monkeypox strains Congo 2003 (DQ011154) and Liberia 1970 (LBR; DQ011156); cowpox virus strains Germany 91–3 (GER91; DQ437593) and Brighton red (BR, NC_003663). Variola virus (VARV) strains: India 1953 (IND53; DQ441428), Brazil 1966 (BRA66; DQ441419), Garcia 1966 (GAR1966; Y16780). CMLV-CMS, camelpox virus strains CMS (AY009089); TATV, taterapox virus (NC_008291), ECTV, ectromelia virus strain Moscow (NC_004105).
